# Dupilumab elicits a favorable response in type-2 inflammatory comorbidities of severe atopic dermatitis

**DOI:** 10.1186/s12948-021-00144-x

**Published:** 2021-06-16

**Authors:** Eustachio Nettis, Lucia Masciopinto, Elisabetta Di Leo, Nicola De Candia, Marcello Albanesi, Danilo Di Bona, Nicola Quaranta, Luigi Macchia

**Affiliations:** 1 Department of Emergency and Organ Transplantation, School of Allergology and Clinical Immunology, University of Bari Aldo Moro, Policlinico Di Bari, Bari, Italy; 2 Section of Allergy and Clinical Immunology, Unit of Internal Medicine – “F. Miulli” Hospital, Acquaviva Delle Fonti, Bari, Italy; 3 Department of Neuroscience and Sensory Organs, Otolaryngology Unit, University of Bari Aldo Moro, Policlinico Di Bari, Bari, Italy

**Keywords:** Monoclonal antibody, Precision medicine, Asthma, Nasal polyps, Rhinoconjuctivitis

## Abstract

**Background:**

This case is the first report describing rapid, successful treatment of severe atopic dermatitis (AD) and comorbid type-2 inflammatory diseases in the same patient, with dupilumab treatment, with no side-effects.

**Case presentation:**

We report on effects of dupilumab in a patient with severe AD, a long-standing history of a mild, perennial allergic rhino-conjunctivitis, moderate asthma and chronic rhinosinusitis with nasal polyps (CRSwNP).

**Conclusions:**

Patients suffering from AD, asthma, allergic rhinitis and CRSwNP may be eligible for dupilumab single treatment that is possibly advantageous also from the pharmaco-economic standpoint.

## Background

Dupilumab is a monoclonal antibody against interleukin (IL)-4 receptor alpha, that inhibits IL-4/IL-13 signalling [1]. The latter cytokines have been implicated in numerous type-2 atopic/allergic conditions, including asthma, atopic dermatitis (AD) and nasal polyposis, often associated as comorbidities [2, 3]. Dupilumab is approved in the USA and in the European Union for patients aged 12 years or older with moderate-to-severe AD and in many Countries for patients with other type-2 inflammatory diseases [3].

## Case presentation

Here, we report on effects of dupilumab in a patient with severe AD, a long-standing history of a mild, perennial allergic rhino-conjunctivitis, moderate asthma and chronic rhinosinusitis with nasal polyps (CRSwNP). The patient was a 43-year-old Caucasian woman who had suffered from AD since early infancy and had undergone various treatments, including topical, nasal and inhaled corticosteroids, topical calcineurin inhibitors, antihistamines, leukotriene receptor antagonists, cyclosporin and systemic corticosteroids. She had also undergone surgical excision of bilateral nasal polyps, 10 years before. At the time of presentation, the patient's daily medication consisted of potent topical steroids (clobetasol propionate ointment 0.05% twice a day), desloratadine (5 mg) and combination therapy consisting of inhaled beclometasone (200 μg) and formoterol fumarate (6 μg), two inhalations twice daily. She also had developed 2 severe AD exacerbations, requiring systemic corticosteroids during the 4 months before starting dupilumab treatment.

## Methods

AD and type-2 comorbidities were evaluated with a full battery of tests, including: the Eczema Area and Severity Index (EASI); SCOring AD (SCORAD); Investigator's Global Assessment (IGA); peak scores on the pruritus numerical rating scale (NRS) and on the sleep NRS during the past 7 days; Patient-Oriented Eczema Measure (POEM); Dermatology Life Quality Index (DLQI); spirometry, Asthma Control Test (ACT); 5-question Asthma Control Questionnaire (ACQ-5); Standardised Asthma Quality of Life (QoL) Questionnaire (AQLQ[S]); 22-item Sino-Nasal Outcome Test (SNOT-22); endoscopic nasal polyp score (ENPS), assessed for each nostril, separately by endoscopy and graded on polyp size, yielding scores from 0 to 4; Loss of Smell Score; Rhinosinusitis Disease Severity; Rhinoconjunctivitis QoL Questionnaire (RQLQ); and complete ear, nose and throat examinations. Skin prick tests (SPT) were performed with common inhalants. Total IgE and a peripheral blood eosinophil count were also detected.

**Fig. 1:**
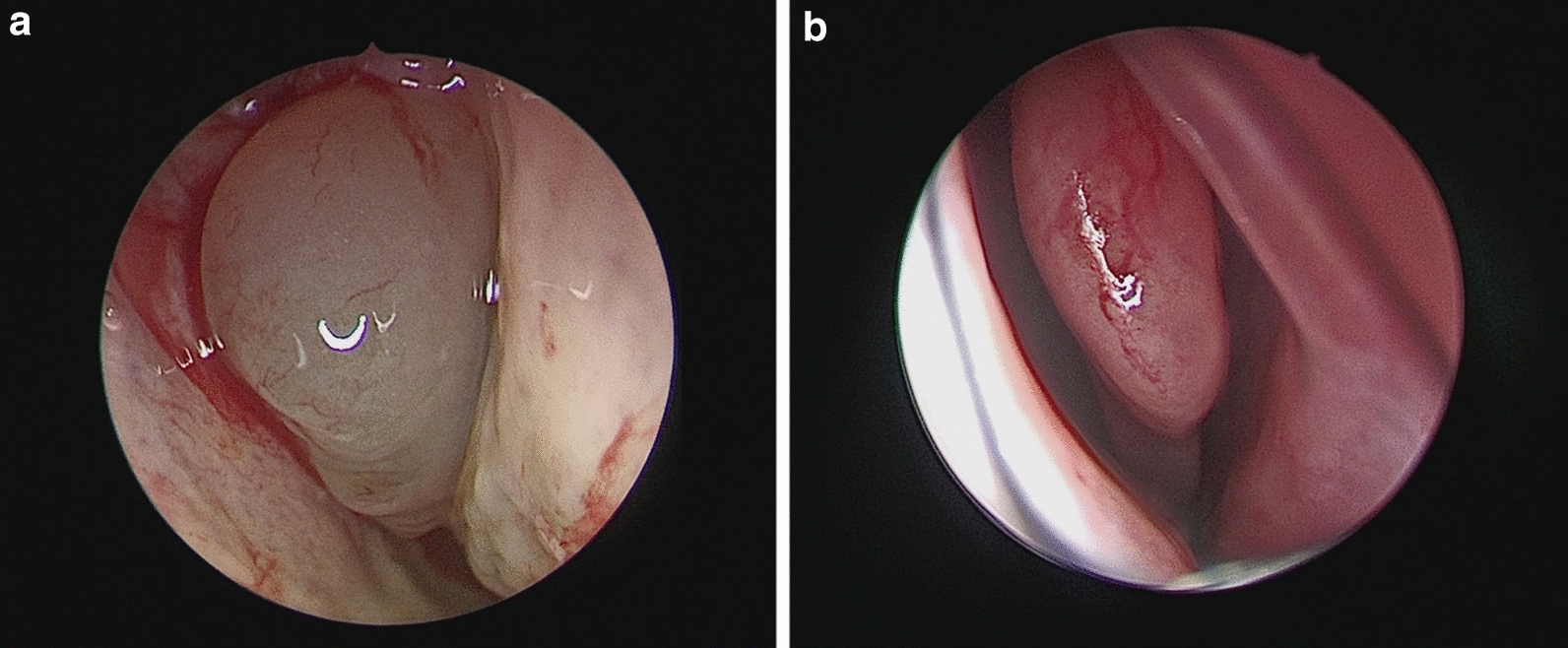
**a** Endoscopic Nasal Polyp Score at baseline for the right side = 2 points; **b** Endoscopic Nasal Polyp Score at week 16 for the right side = 0 points

Dupilumab was administered with a 600-mg loading dose followed by a 300 mg dose every 2 weeks, to control symptoms. The patient underwent follow-up examinations at weeks 4, 8, 12 and 16, after starting dupilumab therapy. During the follow-up period, the patient was asked to continue her pre-treatment therapy. Clinical characteristics of the patient at baseline and during dupilumab treatment are presented in Table 1. At the last follow-up assessment, 16 weeks later, dupilumab had resulted in reduction in EASI score (4 points vs 29), SCORAD score (16 points vs 78) and IGA score (2 points vs 4), indicating a clinically relevant improvement. In addition, dupilumab reduced patient-reported symptoms of AD and its effects on QoL, sleep and pruritus. In fact, for both the POEM and DLQI scores, a reduction in the total score by 19 and 12 points, respectively, was recorded. By week 16, an improvement of 7 points in the peak score for pruritus NRS and 8 points for the sleep NRS had occurred. There were no significant changes in spirometry, ACT, ACQ-5 and AQLQ. The ENPS at baseline was 1 on the left side (1 = small polyps in the middle meatus not reaching below the inferior border of the middle turbinate) and 2 on the right side (2 = polyps reaching below the lower border of the middle turbinate) (Fig. 1a). After 16 weeks, her ENPS was 0 on both the right side (Fig. 1b) and the left side. Furthermore, at week 16 substantial improvements in the total SNOT-22 score (11 points vs 20), in the loss of smell score (2 points vs 1), in Rhinosinusitis Disease Severity assessed by visual analog scale (6 points vs 2) and in the RQLQ score (2.31 points vs 2.85 points) were observed. Moreover SNOT-22 and RQLQ items are frequently rated by patients with sino-nasal disorders as important items affecting their health and QoL. SPT were positive for cat epithelium and house dust mites and clinically relevant. Our patient was able to discontinue topical corticosteroids and showed a reduction in the use of antihistamines and asthma combination therapy throughout the 16-week treatment period (Table 1). She no longer took systemic corticosteroids. Moreover no dupilumab-related side effects were reported.

**Table 1:** Clinical characteristics of the patient at baseline and during dupilumab treatment (with concomitant medications)

	Basal	4 week	8 week	12 week	16 week
EASI score (scale 0–72)	29	12.5	4.6	4	4
SCORAD-total score (scale 0–103)	78	40.9	45.5	20	16
IGA score (scale 0–4)	4	3	3	2	2
NRS pruritus score (scale 0–10)	8	3	8	2	1
NRS sleep score (scale 0–10)	9	1	8	1	1
POEM score (scale 0–30)	21	2	20	2	2
DLQI score (scale 0–28)	13	15	7	1	1
FEV1 (L)	2.60	2.48	2.5	2.5	2.72
FEV1 (% predicted)	89.4%	84%	85%	85%	93.3%
ACT score (scale 0–25)	21	24	25	25	25
ACQ-5 score (scale 0–6)	0.9	0.7	0.1	0.0	0.0
AQLQ score (scale 0–7)	6.3	6.6	6.8	6.7	6.7
Bilateral ENPS (scale 0–8)	3	–	–	–	0
SNOT-22 score (scale 0–110)	20	14	12	15	11
Loss of smell score (scale 0–3)	2	2	1	1	1
Rhinitis disease severity (visual analog scale 0–10 cm)	6	5	3	3	2
RQLQ (scale 0–6)	2.85	2.64	2.51	2.64	2.31
Total IgE levels, KUA/L	1633 kUA/L	–	–	–	901.8 kUA/L
Blood eosinophil Count, cells/μ	239/mm3	–	–	–	50/mm3
Concomitant medications	Clobetasol proprionate oinment 0.05%, 2 times a day; Desloratadine 5 mg/die; Beclomethasone/formoterol fumarate (200/6 μg)two inhalations twice daily	Desloratadine 5 mg as needed; Beclomethasone/formoterol fumarate (200/6 μg) one inhalation twice daily	Desloratadine 5 mg as needed; Beclomethasone/formoterol fumarate (100/6 μg) one inhalation twice daily	Beclomethasone/formoterol fumarate (100/6 μg) one inhalation twice daily	Beclomethasone/formoterol fumarate (100/6 μg) one inhalation as needed

ACQ-5, Asthma Control Questionnaire (5 items); ACT, Asthma Control Test; AQLQ (S), Asthma Quality of Life Questionnaire (standardized version); DLQI, Dermatology Life Quality Index; EASI, Eczema Area and Severity Index; ENPS, Endoscopic nasal polyp score; FEV1, Forced Expiratory Volume in 1 s; IGA, Investigator’s Global Assessment; NRS, Numerical Rating Scale; POEM, Patient-Oriented Eczema Measure; RCSS, Rhinitis Control Scoring System; RQLQ, Rhinitis Quality of Life Questionnaire; SCORAD, Scoring Atopic Dermatitis; SNOT-22, 22-item Sino-Nasal Outcome

Finally, a substantial reduction in peripheral eosinophil count was observed (Table 1).

AD, a common chronic pruritic inflammatory skin disease, has a strong impact on patients' QoL [4]. Comorbidities, such as allergic rhinitis, asthma and CRSwNP are reportedly associated with a reduced health-related QoL [5]. Several studies have shown an important role of type-2 immunity in the immunopathology of AD and related comorbidities [1–3, 6]. The keystone cytokines in type-2 immune response include: IL-4, IL-5, IL-9 and IL-13 [7, 8]. In recent years, the development of therapies against targeting these cytokines has represented a significant advance in the treatment of these diseases. Dupilumab is directed against the alpha subunit of the IL-4 receptor, thereby blocking both IL-4 and IL-13 signalling and hence type-2 inflammation [6]. This explains the good response to dupilumab treatment in our patient with associated type-2 inflammatory diseases. Infact, dupilumab proved effective in AD and ameliorated asthma and allergic rhinitis, as indicated by drug consumption, reducing the need for drugs. Notably, dupilumab was effective in reducing nasal polyps, as shown by ENPS (Table 1).

To our knowledge, this case is the first report describing rapid, successful treatment of severe AD and comorbid type-2 inflammatory diseases in the same patient, with dupilumab treatment, with no side-effects. In conclusion, we suggest that patients suffering from AD, asthma, allergic rhinitis and CRSwNP may be eligible for dupilumab single treatment that is possibly advantageous also from the pharmaco-economic standpoint.

## Data Availability

Not applicable.
